# Evaluating the Implications of Varying Bluetooth Low Energy (BLE) Transmission Power Levels on Wireless Indoor Localization Accuracy and Precision

**DOI:** 10.3390/s19153282

**Published:** 2019-07-25

**Authors:** Umair Mujtaba Qureshi, Zuneera Umair, Gerhard Petrus Hancke

**Affiliations:** Department of Computer Science, City University of Hong Kong, Hong Kong, China

**Keywords:** Bluetooth Low Energy (BLE), Internet of Things (IoT) applications, Wireless Indoor Localization, position estimation, Low Pass Filter, Kalman Filter, localization accuracy and precision

## Abstract

Bluetooth Low Energy (BLE) based Wireless Indoor Localization System (WILS) with high localization accuracy and high localization precision is a key requirement in enabling the Internet of Things (IoT) in today’s applications. In this paper, we investigated the effect of BLE signal variations on indoor localization caused by the change in BLE transmission power levels. This issue is not often discussed as most of the works on localization algorithms use the highest power levels but has important practical implications for energy efficiency, e.g., if a designer would like to trade-off localization performance and node lifetime. To analyze the impact, we used the established trilateration based localization model with two methods i.e., Centroid Approximation (CA) and Minimum Mean Square Error (MMSE). We observed that trilateration based localization with MMSE method outperforms the CA method. We further investigated the use of two filters i.e., Low Pass Filter (LPF) and Kalman Filter (KF) and evaluated their effects in terms of mitigating the random variations from BLE signal. In comparison to non-filter based approach, we observed a great improvement in localization accuracy and localization precision with a filter-based approach. Furthermore, in comparison to LPF based trilateration localization with CA, the performance of a KF based trilateration localization with MMSE is far better. An average of 1 m improvement in localization accuracy and approximately 50% improvement in localization precision is observed by using KF in trilateration based localization model with the MMSE method. In conclusion, with KF in trilateration based localization model with MMSE method effectively eliminates random variations in BLE RSS with multiple transmission power levels and thus results in a BLE based WILS with high accuracy and high precision.

## 1. Introduction

**Figure 1:**
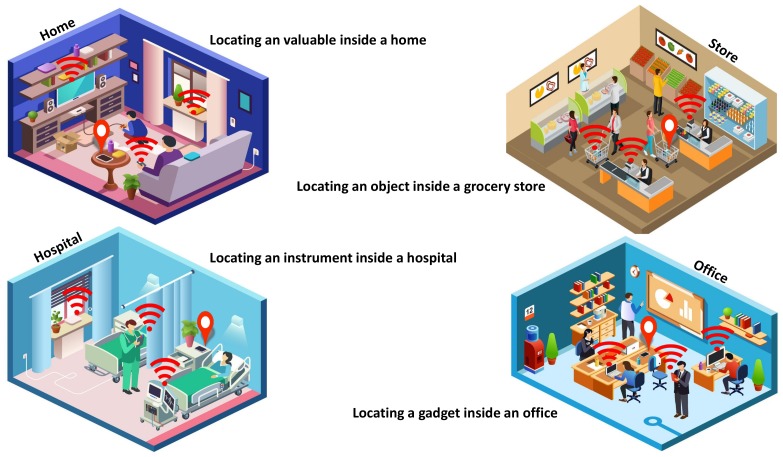
Pre-deployed BLE based IoT Network in Different Indoor Environments.

Internet of Things (IoT) is becoming rapidly ubiquitous with Bluetooth Low Energy (BLE) as its core wireless network technology [1]. BLE also called BLE smart offers very interesting features such as low power consumption and cost, long-range and battery life, small size and portability and secure and simple efficient communication protocols [2,3]. Such features have allowed BLE to be widely embedded in common consumer electronic devices and to be an integral part of smart gadgets (such as smartphones, smartwatches and, laptops, etc.). These BLE based wireless devices intercommunicate with one another and form an IoT network that provides different services and applications to people in outdoor as well as indoor environments [4]. As people spend a substantial proportion of their time in indoor environments, the interaction of smart devices and gadgets interaction provide the basis for many novel IoT applications, such as indoor positioning, object tracking and indoor navigation, home automation, health monitoring, proximity-based advertisement and retail marketing [5,6,7]. The extensive use of BLE devices in IoT applications has laid the foundation of a virtual BLE infrastructure in different kinds of indoor environments such as buildings, offices, homes and shops [8]. For example in Figure 1, BLE based IoT network deployed in a home can be used to locate people, valuables or objects of interest [9]. A pre-deployed BLE based IoT network in an office building can help the company to maintain the time records of its employees [10]. Whereas, a BLE based IoT network installed in a retail shop can be used to provide information on the time spent by different customers in front of different items of their interest [11]. Similarly, a BLE based IoT network deployed in hospitals can be used to provide critical information about the daily routines of patients and can help to develop performance indicators to maintain a healthy environment [12]. In all of these applications, the location of the target (human, object or robot) is of prime importance and to estimate the coordinates is a challenging task. In this context, the pre-deployed BLE IoT network can be exploited to form Wireless Indoor Localization System (WILS) that can provide strategic information of the targets, thus augmenting the use of existing applications [13].

In light of the motivation presented above, BLE based WILS can be formed by smart use of the existing BLE based IoT network infrastructure in different indoor environments. Such a BLE based WILS would be a low-cost system, as it will only consist of the pre-deployed BLE devices. At the same time, the system is simple in use because the Received Signal Strength (RSS) of the BLE devices is used to derive the location(s) of the different target(s) with the use of suitable signal based localization algorithms. Wireless signal based localization algorithms derive absolute or relative coordinates of the target through the RSS of the devices in an indoor environment such as proximity [8], fingerprinting [14] and trilateration [15,16]. Proximity algorithm only indicates an approximate presence of the target(s) (also referred to as a tag(s)) in reference to a known location of a BLE transmitting device(s) (also referred to as an anchor(s)) [17]. Whereas wireless fingerprinting requires the matching of pre-recorded RSS or fingerprints of the BLE anchors with the RSS recorded by the target for localization within the indoor environment that introduces unnecessary off-line workload before matching of the online fingerprints to locate the target [18]. Unlike proximity and fingerprinting, the trilateration algorithm estimates the absolute or relative location of the target with reference to the locations of (at least 3) BLE devices [19]. It uses a path loss model to derive the distance from the corresponding RSS samples. Trilateration algorithm is widely used for its simplicity and ability to calculate the target location(s) from the RSS of the pre-deployed BLE devices.

The efficacy of the WILS depends on the localization accuracy and location precision. The localization accuracy of the WILS defines the measure of correctness of the estimated location of the target [20,21,22]. Whereas, the location precision of the WILS defines the percentage of the estimated location of the target of a certain localization accuracy sustained for the defined interval of time [20]. To design a low-cost BLE based WILS with a high localization accuracy and high precision is a key requirement in enabling IoT applications but it is equally challenging and difficult. Since RSS is used to locate the target, the localization accuracy and precision depends on the stability of the BLE RSS, which in fact, depends on the transmission power used by the BLE devices. As the BLE wireless standard allows BLE devices to operate at multiple transmission power levels, it is intuitive that at a high transmission power level, BLE RSS is more stable compared to BLE RSS at a low transmission power level [23,24]. This issue is not often discussed as most works on localization algorithms use the highest power levels but it has important practical implications for energy efficiency, e.g., if a designer would like to trade-off localization performance and anchor’s battery lifetime. Furthermore, BLE RSS is susceptible to fast fading noise [14] and multi-path effects [25] caused by clutter present in the indoor environment, regardless of the transmission power level used by the wireless devices [26]. There are other factors such as the effects of the non-linear amplifiers of the BLE devices, the antenna gain variation of the signal transmitters and different kinds of antennae used by various vendors [27]. But their effect is of constant proportion in comparison to the effects of fast fading noise [28], the change in transmission power levels [29] and interference level of multi-paths [23,24]. Regardless of the gain, antennae and device heterogeneity, the random variation persists in every BLE signal mainly due to its low energy characteristic. A significant BLE RSS variation occurs when devices operate from high to low transmission power levels. As such, we investigate the extent of localization degradation when we use lower transmission power levels.

Therefore, the focus of this paper is to investigate the implications of BLE signal variation due to the use of multiple transmission power levels on WILS. To do so, we use the well-known and established trilateration based localization with Centroid Approximation (CA) method and Minimum Mean Square Error (MMSE) method. The trilateration based localization model is used to analyze the BLE RSS variations. The RSS variation causes the WILS to deviate, that results in low localization accuracy and low localization precision. To address this problem, we further investigate two different filters i.e., a Low Pass Filter (LPF) and Kalman Filter (KF) with trilateration based localization model and analyze their performance in eliminating the random variations from BLE RSS at multiple transmission power levels. Furthermore, we compare the performance of the two filters in terms of localization accuracy and localization precision with BLE based WILS operating at multiple transmission power levels. Thus, the contributions of this paper are as follows:

- We investigate the problem of BLE RSS variation caused by the multiple transmission power levels in a BLE based WILS.
- We evaluate the effects of BLE RSS variation on localization accuracy and localization precision of the WILS.
- We further investigate and compare the performance of LPF and KF to improve localization accuracy and localization precision.
- Lastly, we evaluate the implications of lower power levels on WILS which is practically useful for realizing the trade-off between accuracy along with precision and the device lifetime when deploying energy efficient WILS where lower power transmission levels are used.

The rest of the paper is organized as follows. Section 2 reviews the relevant work present in the literature and discuss how is our work different from others. In Section 3, we present the concept of BLE based WILS along with the set of assumptions considered to investigate the problem of BLE RSS variation with multiple transmission power levels. In Section 4, we briefly explain the trilateration based localization model and discuss the limitations of the BLE based WILS. In Section 5, we explain in detail the extended localization model in which two filters i.e., LPF and KF, are introduced in a simple trilateration based model. Section 6 presents the details on the set of experiments and discusses the results. Finally, Section 7 concludes the paper and presents future work.

## 2. Related Work

BLE is widely adopted as the de facto wireless standard for IoT applications as it continues to progress to meet their requirements [30]. The evolution from the classical Bluetooth wireless technology to the BLE has been extensively discussed in [31], along with the discussion on its features, specification, use in existing IoT applications [8] and its viability in future IoT applications in light of the different commercial and noncommercial BLE systems present in the market [11]. BLE features [2] such as low power consumption and cost, long-range and battery life, secure and fast efficient communication and small size and portability, allows BLE to be tagged to any entity (stationary or moving) in comparison to other wireless standards (such as WiFi [32], RFID [33], UWB [34], Infrared [35] and Ultrasound [36]) that rely more on a static network [37]. The existing IoT applications have already laid the foundation of a BLE based IoT network infrastructure that can be used for applications such as micro-positioning and indoor localization, to make the existing applications smarter [38]. The foresight that the pre-deployed BLE based IoT network infrastructure for sensing, monitoring and control IoT applications in different indoor environments can be used for additional applications, such as BLE based WILS.

In the context of WILS, BLE has been extensively studied and evaluated for target localization in indoor environments [27,39,40,41]. These studies mainly focus on analyzing the impact of the different parameters such as scanning window, transmission interval, number of frequency channels, the orientation of the devices, different indoor structures, device density and presence of line of sight (LOS) [42] and Non-Line of Sight (NLOS) with different objects [41] over BLE RSS in an indoor environment. For target localization, BLE is used with different approaches to estimate the location, such as wireless signal based localization algorithms, e.g., proximity [15], fingerprinting [14] and trilateration techniques [43]. Some researchers have proposed alternative approaches such as geometric methods [17], machine learning algorithms e.g supervised learning [44,45] and unsupervised learning [46] and crowdsourcing approaches [47]. In reference to the preferable range of localization accuracy for different targets in an indoor environment, Dahlgren et al. [48] evaluated BLE RSS for indoor localization to achieve localization accuracy within the range of 2 m to 5 m (i.e., <2 m, <3 m, <4 m, <5 m and >5 m) with different commercial BLE commercial systems and in different indoor environments such as offices, corridors and rooms, etc. The authors conclude that the BLE RSS variations caused by the noise effects that are induced by the clutter present in the indoor environment to be the prime reason that effects the localization accuracy and reported maximum localization accuracy achieved was approximately 3 m.

To deal with the BLE RSS variation, a filter based approach is the most common method to address the randomness in wireless signals. Researchers have proposed different filters, such as moving average filter [49], median filter [14], LPF [49], KF [50], extended-KF [51] and particle filter [52], along with trilateration algorithms. In [53] the authors used filter based approach for localization and reported a localization accuracy less than 1 m by using a KF with trilateration. In [54] the authors used the channel diversity of the BLE signals for indoor localization. The authors used KF to remove the fluctuations from the RSS of the BLE devices and reported a localization accuracy of less than 1 m with trilateration technique however all experiments conducted were on default transmission power setting.

In comparison to filter based approach, a number of researchers have proposed non-filter based approach in which the multiple transmission power level feature of BLE is analyzed in context of reducing the noise effects that causes the fluctuations in RSS. A detailed study is presented in [24] in which the authors have analyzed the effects of multiple transmission power levels on BLE RSS. The authors show the presence of multi-path fading effects at different transmission power levels and propose machine learning algorithms such as Support Vector Machines and K-Nearest Neighbour algorithms along with wireless fingerprinting technique with BLE based WILS for indoor localization. In [23], the authors also proposed a multiple transmission power level feature to address multi-path effects in BLE RSS. From their experiments the authors show that at low transmission power level, the effects of the multi-paths is low. The reason being that at low transmission power level, the majority of the multi-paths fades away while propagating towards the destination node. In the experiments, the authors attached the BLE devices to a ceiling that operates with two transmission power levels, i.e., −30 dBm and −42 dBm, which is in LOS with a laptop at a distance of approximately 2 m, showing that the RSS is more stable and the estimated localization error is 1 m. The experimental setup of the work in [23], is highly impractical in a real-world scenario and the RSS of BLE at such low transmission power levels can hardly be recorded beyond a 1 m distance.

Recently, multiple transmission power level is used with filter-based approach. The authors in [28] explored different transmission power levels (i.e., 4 dBm, 0 dBm, −4 dBm and −8 dBm) and studied their effect on RSS with distance. The authors used a K-Nearest Neighbour (KNN) based fingerprinting technique and four different filters to smooth the RSS. The authors reported the result with a maximum localization accuracy of 2.9 m when all devices operate at the highest transmission power level i.e., 4 dBm. Golestanian et al. in [55] exploited and proposed multiple transmission power level feature of BLE based WILS to deal with unreliability of the RSS. The authors analyzed the variation of BLE RSS at −4 dBm, −8 dBm, −12 dBm and −20 dBm and used moving average filter to remove the variation in the RSS which ultimately reduces the error in distance estimation.

The works presented in [24,28] motivated us to conduct a comprehensive evaluation of a BLE based WILS by exploring the multiple power transmission feature for target localization in an indoor environment. In [56], we analyzed a BLE based WILS with multiple transmission power levels and used a trilateration algorithm for target localization. We conducted a set of experiments in a classroom environment and estimated the location of the 3 targets in the presence of LOS and NLOS. Out of 5 transmission power levels, the maximum localization accuracy achieved was 2.2 m when all BLE devices operated at 10 dBm, which is the highest transmission power level, and localization accuracy of 5 m with −8 dBm, which is the lowest transmission power level. The reason is that BLE devices operate at low transmission power levels, hence the RSS gets weak and attenuated due to multi-path effects and clutter present within the environment. The reported results were achieved by using the average of the collected raw RSS samples. The studies [49,54] provided the result that as high as a 1 m localization accuracy can be achieved with the use of the filters with BLE by using a trilateration algorithm. To the best of our knowledge, the majority of the researchers have addressed the problem of RSS variation at default transmission power level or at higher transmission power levels with filters to achieve high localization accuracy. The problem of RSS variation caused by the change in transmission power levels in BLE based WILS localization accuracy and precision has not been highlighted and addressed.

Localization accuracy and localization precision are two important parameters in WILS. It is important that the system should be able to sustain the high localization accuracy with high location precision. Most of the researchers calculate the mean localization error derived from the average of the RSS sample collected. However, for real-time localization, the use of instantaneous samples is also preferred. But, instantaneous RSS samples are erroneous, such data is usually averaged to derive an approximate value. By the use of a filter, the erroneous samples can be removed to result in a smooth RSS. To the best of our knowledge, the majority of the researchers have addressed the problem of RSS variation at the default transmission power level. In this paper, we comprehensively highlight the problem of RSS variation with change in multiple transmission power levels and propose a simple solution to address the highlighted problem.

## 3. Bluetooth Low Energy (BLE) Based Wireless Indoor Localization System (WILS)

**Figure 2:**
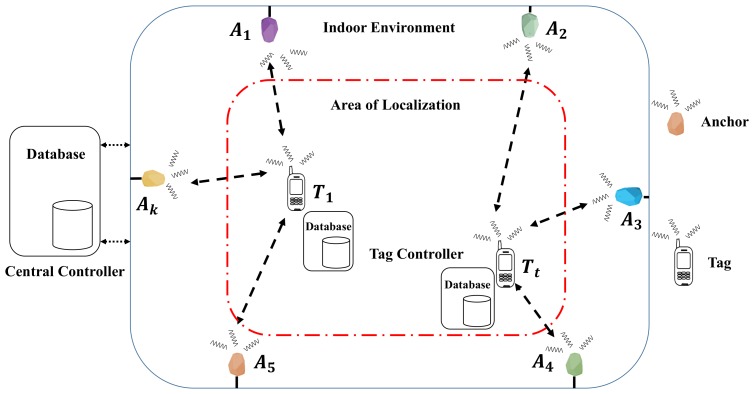
BLE based WILS.

In this section, we shall explain the generic model of BLE based WILS. Generally, a WILS consists of a set of pre-deployed access points or anchors, denoted by $A_{k}\in A$, where *k* represents the *k*th anchor and a set of targets or tags, denoted by $T_{t}\in T$, where *t* represents the *t*th tag in an indoor environment as shown in Figure 2. The location of the anchors, denoted by ($x_{A_{k}},y_{A_{k}}$) is fixed and known. Whereas, the tag location, denoted by ($x_{T_{t}},y_{T_{t}}$), needs to be estimated in an area referred to as Area of Localization (AoL). In a typical BLE based WILS both the anchors and tags are wireless devices, embedded with BLE wireless transceivers that allow them to operate in the 2.4 GHz license-free band. In a BLE based system, 2.4 GHz ISM band is divided into 40 BLE channels, each 2 MHz [14]. Out of the 40 BLE channels, 3 channels (i.e., 37, 38 and 39 of the BLE 2.4 GHz ISM band) are designated as advertising channels, while the remaining 37 are communication channels [14]. The BLE wireless standard allows each BLE device (i.e., anchor and tag) to be identified by a Universal Unique Identifier (UUID) [57]. The UUID is advertised in small data packets called beacons, by hopping pseudo-randomly between 3 advertising BLE channels. The rate at which the beacons are advertised is defined by an advertising interval, denoted by $\tau_{adv}$, that ranges approximately from 2000 ms to 100 ms [57]. A large advertising interval corresponds to low beaconing whereas a small advertising interval corresponds to high beaconing. Each anchor has the capability to transmit the beacons at multiple transmission power levels [25]. These transmission power levels are listed in [56] and shown in Table 1. The transmission power level is denoted by $\rho_{l}$, where *l* is the index of the power level as listed in Table 1. The transmission power level defines the transmission range of the device. High transmission power corresponds to a large transmission range and vice versa. We assume at a time instance τ all anchors of the anchor set *A* operate at similar transmission power levels $\rho_{l}^{\tau }$. With that what we mean is, at a time instance τ, all anchors will operate at a single transmission power level, i.e., $\rho_{l}^{\tau }$. Any BLE device that resides within the range of an anchor can scan for their beacons if they are advertised. The rate of scanning beacons is defined by the scanning interval [57], denoted by $\tau_{scan}$. A large scanning interval allows many beacons to be collected, whereas a small scanning interval allows less beacons to be collected. Generally, $\tau_{scan}=$ 1 s, is the default scanning interval which is adopted universally and a $\tau_{adv}=$ 250 ms is the most optimal setting considering scanning interval $\tau_{scan}=$ 1 s. It is assumed that the tag is in Line of Sight (LOS) and resides within the transmission range of at least 3 anchors at all times.

In a typical BLE based WILS, all these anchors are further wired with a central controller, that hosts a central database, where the data reported by each anchor is stored and processed to estimate the location of the tag. Similarly, a tag also hosts a tag controller and associated with the tag controller is its database in which the tag controller stores and process the data that it receives from the anchors. The data here is basically the raw RSS of the BLE devices, e.g., if the anchors are residing within the transmission range of the tag, the anchors report to the central controller with the RSS of the tag and the controller updates the data stored in its database. Similarly, the tag reports to its controller with the RSS of the anchors and the tag controller updates the data stored in its database. Whenever a target arrives in the range of 3 or more anchors, the central controller or the tag controller, or both, can initiate the process of localization and estimate the location of the tag. The entire system is shown in the Figure 2 and the symbol along with their definitions used in the model are listed in Table 2.

The initiation of the localization process can be triggered by the anchor or by the tag or both. If a tag is a lost valuable, the localization process is initiated by anchors and the central controller, whereas if the person wants to know its location inside a building the person triggers the localization process through his/her cell phone that acts as a tag. BLE devices are resource constrained i.e., they have limited computational power, memory and limited battery life.

The multiple transmission power level features of BLE devices allow them to operate from higher to lower transmission power. The main advantage of operating at a high transmission power level is that the BLE signal is less susceptible to noise effects at a significantly larger distance. At high transmission power levels, two BLE devices (central-peripheral roles) are able to pair in a very short time interval (in order of 1–2 ms) because of low Bit Error Rate (BER) and low latency in the link between the two devices. As a disadvantage, the continuous transmission at high transmission power levels drains the battery of the BLE devices that ultimately leads to the short lifespan of the device. The advantage of operating at low transmission power increases the overall lifespan of the BLE devices but the ability of BLE devices to transmit at large distances is restricted. At low transmission power levels, the BLE signal is prone to noise effects that result in high BER and link latency which increases the overall connection interval and pairing time between the two BLE devices [26]. In the context of wireless localization, transmission power plays an important role in localization specifically in indoor environments. The high transmission power leverages the coverage area of an individual device therefore, fewer devices can be used to locate a large number of targets in an AoL with a reasonable localization accuracy and precision at the cost of battery lifetime. Whereas, operating at low transmission power levels increases the battery lifetime but restricts the coverage area that demands a significant number of devices to be deployed to cover an AoL and locate the targets with a reasonable localization accuracy and precision. In this regard, the researchers have devised transmission power management schemes to optimize the energy efficiency and overall battery lifetime of the BLE devices [58,59,60]. Regardless of the transmission power management schemes, the change in the transmission power affects the BLE RSS. This change in the RSS will affect the overall localization accuracy and localization precision of the WILS. Because at low transmission power levels, RSS is highly unstable due to multi-path effects and clutters present in the indoor environment. In the next section, we present the set of techniques to estimate the localization system and investigate the effect caused by the change in multiple transmission power levels over localization accuracy and localization precision.

## 4. Trilateration Based Localization Model

**Figure 3:**
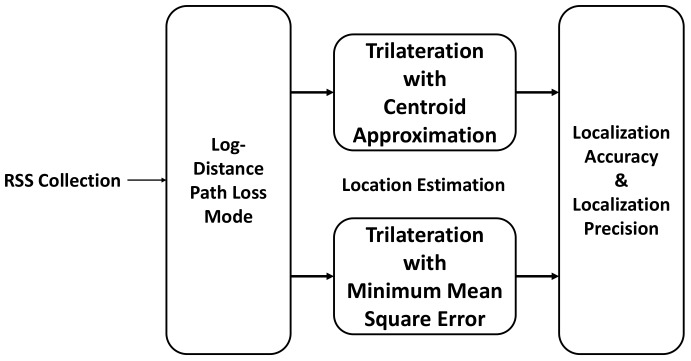
Trilateration Based Localization Model.

In BLE based WILS, we adopt a simple trilateration based localization model. The stepwise execution of each technique is shown in Figure 3. Initially, the tag records the RSS of the anchors by receiving the beacons from the set of anchors in whose range the tag resides. The RSS of the beacon signal decreases with an increase in distance between the anchor and tag [24]. This relation is quantified and translated to estimate the distance from the tag to each anchor present in the vicinity. To translate the RSS into the distance and to estimate the location of the tag, the tag controller uses a path loss algorithm that maps the RSS to corresponding distances. The distance estimated from the path loss algorithm is later used in the trilateration algorithm to estimate the location of the tag. The trilateration algorithm is composed of a system of equations that can be solved in a number of ways. We use the two most common solutions for solving the trilateration system of equations, i.e., Centroid Approximation (CA) and Minimum Mean Square Error (MMSE). The location estimated by both of these algorithms is then used to calculate the location accuracy and localization precision to provide an insight into the efficacy of the BLE based WILS. The entire trilateration based localization model shown in Figure 3 is discussed in detail in the following section.

### 4.1. Log-Distance Path Loss (LDPL) Model

The basic behavior of a wireless signal is that the RSS of the signal decreases with the increase in the distance between a transmitter and a receiver [61]. This decrease in the RSS is commonly known as path loss. Path loss is a measure of the attenuation of a wireless signal that it undergoes when the distance between transmitter and receiver is increased [61]. This relationship between the RSS and distance can be modeled. The Log-Distance Path Loss (LDPL) model is one of the most common methods that is used to map the RSS samples with their corresponding distances [61]. Mathematically, it is represented by Equation (1) [62]:

(1) $$RSS\left(d_{A_{k},T_{t}}\right)=RSS\left(d_{0}\right)-10n\times log\left(\frac{d_{A_{k},T_{t}}}{d_{0}}\right),$$

where, $RSS(d_{A_{k},T_{t}})$ (in dBm) represents the strength of the beacon signal when the tag $T_{t}$ is at a distance $d_{A_{k},T_{t}}$ (in m) from the anchor $A_{k}$, $RSS(d_{0})$ (in dBm) represents the beacon strength at default distance ($d_{0})=$ 1 m and *n* is the attenuation factor that characterizes an indoor environment (typically ranges in 2 to 4) [24]. The distance $d_{A_{k},T_{t}}$ from an anchor $A_{k}$ to the tag $T_{t}$ is calculated through the LDPL model by using Equation (2):

(2) $$d_{A_{k},T_{t}}=10^{\frac{RSS\left(d_{0}\right)-RSS\left(d_{A_{k},T_{t}}\right)}{10\times n}}$$

For the LDPL model, to reliably translate the RSS samples to distance, it is important that the path loss exponent should be selected carefully. Since the indoor environment is not uniform, it is better to estimate the path loss exponent empirically rather than theoretically, i.e., selecting a single value that can be suitable for one environment but may not be the best fit for other environments. Therefore, we used an empirical method to calculate *n* for RSS-distance mapping. Once $d_{A_{k},T_{t}}$ is calculated, the tag initiates the trilateration algorithm.

### 4.2. Trilateration Algorithm

The trilateration algorithm is used to determine absolute or relative locations of a tag with reference to the locations of at least 3 BLE anchors [62]. In a BLE based WILS, we assume the locations of all the deployed anchors are reference locations. The estimated location, denoted by (${\hat{x}}_{T_{t}},{\hat{y}}_{T_{t}}$) of the tag is calculated with respect to the set of reference locations of anchors by using the trilateration algorithm. Mathematically, it is expressed by Equation (3) [63]:

(3) $$\left\{\begin{array}{c} d_{A_{1},T_{t}}=\sqrt{{\left(x_{A_{1}}-{\hat{x}}_{T_{t}}\right)}^{2}+{\left(y_{A_{1}}-{\hat{y}}_{T_{t}}\right)}^{2}}\\ d_{A_{2},T_{t}}=\sqrt{{\left(x_{A_{2}}-{\hat{x}}_{T_{t}}\right)}^{2}+{\left(y_{A_{2}}-{\hat{y}}_{T_{t}}\right)}^{2}}\\ .\\ .\\ d_{A_{k},T_{t}}=\sqrt{{\left(x_{A_{k}}-{\hat{x}}_{T_{t}}\right)}^{2}+{\left(y_{A_{k}}-{\hat{y}}_{T_{t}}\right)}^{2}} \end{array}\right.$$

The estimated location coordinates of the tag are calculated by solving the system of equations given by Equation (3) [63]. There are a number of solutions present in the literature to solve these equations. However, the two common methods that are widely used are discussed.

#### 4.2.1. Centroid Approximation (CA)

Centroid approximation is one of the simplest solutions of the trilateration algorithm [64]. This method works reasonably well in an indoor environment when the anchors are in LOS with the target. It uses simple distance approximation or distance scaling between the reference locations of the selected anchors and the tag for localization. Mathematically, it is given as Equation (4) [64]:

(4) $${\hat{x}}_{T_{t}}=\frac{\sum_{k=1}^{\left|k\right|}\frac{x_{A_{k}}}{d_{A_{k},T_{t}}}}{\sum_{k=1}^{\left|k\right|}\frac{1}{d_{A_{k},T_{t}}}},{\hat{y}}_{T_{t}}=\frac{\sum_{k=1}^{\left|k\right|}\frac{y_{A_{k}}}{d_{A_{k},T_{t}}}}{\sum_{k=1}^{\left|k\right|}\frac{1}{d_{A_{k},T_{t}}}}.$$

Here, $1/d_{A_{k},T_{t}}$ is the weight assigned to the coordinates of the tag. Trilateration based on CA is simple to implement as it estimates the location of the tag only once and with each iteration a new estimation is calculated that is independent of its previous estimation.

#### 4.2.2. Minimum Mean Square Error (MMSE)

Minimum Mean Square Error (MMSE) is another common solution used to solve the set of equations in the trilateration algorithm. This method uses the matrix approach in which the system of equations as shown in Equation (3), is converted into a matrix and solved by the MMSE method [65]. Mathematically, it is a given as Equation (5) [65]:

(5) $$\begin{array}{c} \begin{array}{c} SX=b,\\ X={\left(S^{\prime }S\right)}^{-1}S^{\prime }b, \end{array} \end{array}$$

where, *S* is a matrix that consists of the coordinates of anchors, *b* is also a matrix that consists of tag-anchor distances along with the coordinates of anchors, and *X* is the estimated location (${\hat{x}}_{T_{t}},{\hat{y}}_{T_{t}}$) of tag $T_{t}$.

The CA and MMSE based trilateration algorithms rely on the accurate distance $d_{A_{k},T_{t}}$. The distance $d_{A_{k},T_{t}}$ is an array of distance values translated from the RSS samples of the anchors resulting from the LDPL model. For the trilateration algorithm to be accurate, it is very important that the RSS samples that are translated to their corresponding distances values are as accurate as possible. However, with BLE devices operating at multiple transmission power levels, it is highly likely that as the transmission power decreases, the BLE RSS gets weaker. Therefore, especially at low transmission power levels, the BLE based WILS may result in some errors or deviations in the estimated location. The trilateration based localization model provides the estimated location of the tag. The two important metrics to quantify the feasibility of the BLE based WILS are localization accuracy and localization precision. The localization accuracy and localization precision are discussed in the next section.

### 4.3. Localization Accuracy and Localization Precision

The ultimate goal of the BLE based WILS is to locate the tag with accuracy and high precision. Localization accuracy and localization precision are the two most important metrics that are critical for any localization system. To better understand these two concepts, we first define the two metrics formally.

#### 4.3.1. Localization Accuracy or Localization Error

Localization accuracy is the measure of the correctness of the estimated location of the target by the system [20]. It is denoted by $\varepsilon (T_{t})$. Localization accuracy is computed by estimating the error, that is the difference between the real coordinates of the tag, i.e., ($x_{T_{t}},y_{T_{t}}$) and estimated coordinates of the tag, i.e., (${\hat{x}}_{T_{t}},{\hat{y}}_{T_{t}}$). Mathematically, it is given by Equation (6) [66]:

(6) $$\varepsilon \left(T_{t}\right)=\sqrt{{\left({\hat{x}}_{T_{t}}-x_{T_{t}}\right)}^{2}+{\left({\hat{y}}_{T_{t}}-y_{T_{t}}\right)}^{2}}$$

Here, $\varepsilon (T_{t})$ is the error (in m) in the estimated location with reference to the true location of the tag. A small localization error means that the estimated location is highly accurate, whereas a large localization error means that the estimated location is less accurate.

#### 4.3.2. Localization Precision

Localization precision is the percentage of the accuracy of the estimated location over a period of time or for the recorded stream of RSS samples [20]. Localization precision can be computed using Equation (7) [66]:

(7) $$\sigma \left(T_{t}\right)=\sqrt{\sigma_{{\hat{x}}_{T_{t}}}^{2}+\sigma_{{\hat{y}}_{T_{t}}}^{2}}$$

Here, $\sigma_{{\hat{x}}_{T_{t}}}^{2}=\frac{\sum_{1}^{\left|RSS\right|}{\left({\hat{x}}_{T_{t}}-x_{T_{t}}\right)}^{2}}{\left|RSS\right|}$ represents the deviation in the *x* coordinate of the tag, $\sigma_{{\hat{y}}_{T_{t}}}^{2}=\frac{\sum_{1}^{\left|RSS\right|}{\left({\hat{y}}_{T_{t}}-y_{T_{t}}\right)}^{2}}{\left|RSS\right|}$ represents the deviation in the *y* coordinate of the tag, RSS is the RSS sample vector of 200 samples and $\sigma (T_{t})$ defines the overall deviation in the estimated location of the tag calculated by each sample of RSS. Typically, the value ranges from 0 to 1. A low deviation ($\sigma (T_{t})=0$) means that the resultant localization error is small and does not deviate much, therefore the overall WILS yields a high localization precision. A high deviation means that the resultant localization error is large and it deviates a large proportion over a period of time, ultimately leading to a low localization precision. As different targets require different localization accuracies, at the same time it is equally important that the precision of the localization system should also be reasonably high.

As BLE devices operate at a high transmission power level, the BLE RSS is expected to be more stable and results in reasonable or high localization accuracy with a reasonable or high localization precision. Similarly, at low transmission power levels, the BLE RSS gets more unstable and may result in poor or low localization accuracy and low localization precision. In order to better understand the effect of multiple transmission power levels on localization accuracy and localization precision of a BLE based WILS, we deploy the entire BLE setup and analyze the effect over BLE RSS in the next section.

### 4.4. Limitation of a BLE Based WILS

**Figure 4:**
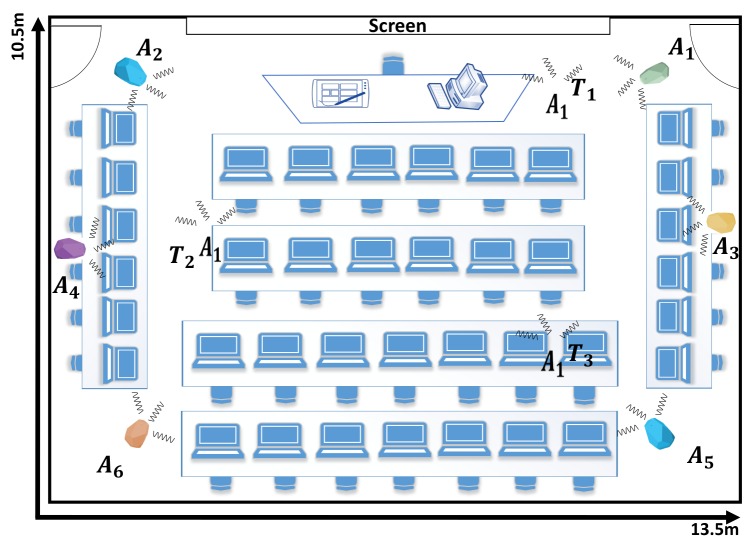
Environment 1: Indoor classroom that is equipped with 6 BLE estimotes used to estimate 3 tag locations.

**Figure 5:**
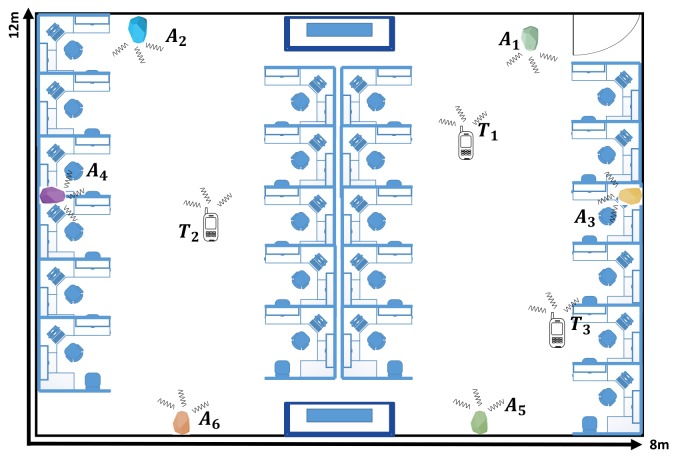
Environment 2: Computer laboratory that is equipped with 6 BLE estimotes used to estimate 3 tag locations.

There are a number of factors that limit the performance of a BLE based WILS and affects the localization accuracy and localization precision. The premier factor is the instability of the RSS. To better understand the effect, consider two different indoor environments as shown in Figure 4 and Figure 5 in which an anchor set *A* consisting of 6 BLE anchors is deployed. Environment 1 is a classroom with dimensions of 13.5 m × 10.5 m and environment 2 is a computer laboratory with dimensions of 8 m × 12 m respectively. Both of these indoor environments represent environments with a complex indoor structure due to the presence of clutters such as cubicles, PCs, tables, chairs and people. The deployed anchors that are used to locate 3 tags at three different locations. The real locations of the anchors and tags are listed in Table 3. The rest of the parameters are in accordance with our assumption made in Section 3. There are a total of 9 different transmission power levels. All tags reside within the transmission range of the BLE anchors, and thus the tags can receive the BLE beacons and report the RSS to the tag controller. Initially, the effect of multiple transmission power is analyzed, later, the location of the tag is computed.

#### 4.4.1. Effects on BLE RSS

To better understand the effects on BLE RSS, we first analyze the BLE RSS for 9 different transmission power levels with respect to distance. For analysis, we calculated the average RSS and RSS variation of the BLE sample vector that consists of 1000 raw RSS collected at a distance of 1 m to 10 m with the step size of 1 m between the BLE transmitter and receiver within an AoL of environment 1 and environment 2.

**Figure 6:**
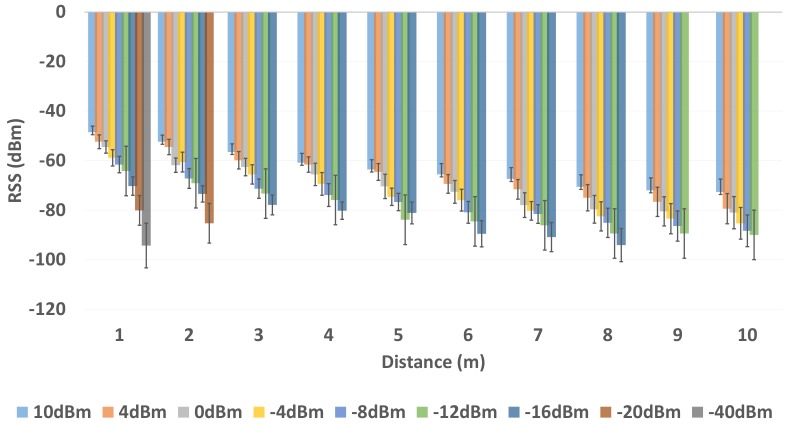
BLE RSS (dBm) with Multiple Transmission Power Level in Environment 1.

**Figure 7:**
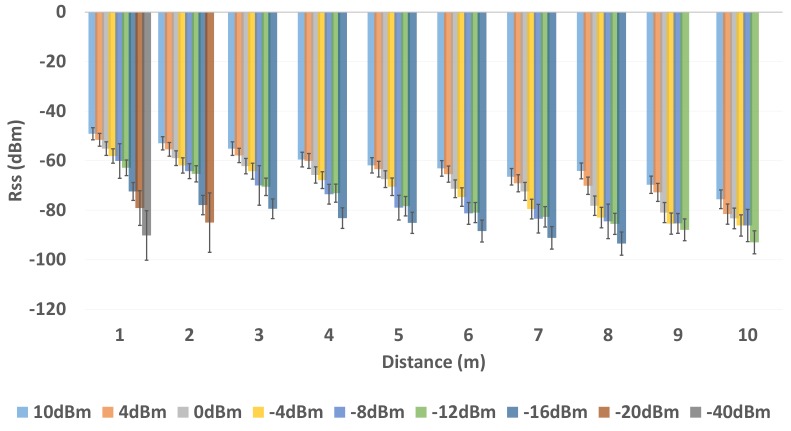
BLE RSS (dBm) with Multiple Transmission Power Level in Environment 2.

Figure 6 and Figure 7 show the average BLE RSS along with variation in BLE RSS at distance that ranges from 1 m to 10 m with a step size of 1 m. There are two important phenomena to be observed in Figure 6 and Figure 7. First, the average BLE RSS is different for 9 different transmission power level configurations. It can also be noticed that the average BLE RSS decreases with increase in the distance between transmitter and receiver as shown in Figure 6 and Figure 7. Due to the difference in the transmission power levels (i.e., approximately 4 dBm), the average RSS at each transmission power level is different. The decrease in the average RSS with respect to distance is due to the fact that BLE signal is attenuated with distance. Second, for each transmission power level configuration, the BLE RSS exhibits a certain amount of variation. The BLE RSS variation tend to increase with increase in distance between the transmitter and receiver. Because of the difference in signal strength, the RSS variation is also different for each transmission power level with respect to distance. In Figure 6 and Figure 7 it can be clearly observed that at each subsequent unit distance, the attenuation in BLE RSS and RSS variation is more in comparison to the BLE RSS and RSS variation measured at the previous unit distance. It is worth noticing that, from the unit distance of 2–3 m, the BLE devices that operate with the transmission power levels from 10 dBm to −12 dBm, the RSS is significantly attenuated and RSS variation is severely increased with respect to distance. Whereas, for BLE devices that operate with transmission power levels of −12 dBm to −40 dBm, the RSS attenuation and RSS variation is much more, with respect to distance. Thus, these results clearly show that along with the difference in the RSS attenuation there is a considerable difference in the BLE signal variation at all 9 different BLE transmission power levels with respect to distance.

Based on the transmission range feasibility (such as severe RSS attenuation, abnormal signal variation and limited coverage) with respect to our assumptions for localization within the two environments, 5 transmission power levels (i.e., 10 dBm, 4 dBm, 0 dBm, −4 dBm, −8 dBm) are selected. Path loss exponent is also an important factor that help environment characterization with wireless signals. In order to have an insight of the two indoor environments, we calculate the path loss exponents empirically by using a linear curve fitting method as discussed in [65] for each transmission power level that is now selected for localization. This is important because when the LDPL model is applied, the RSS-Distance translation can be accurate. The path loss exponents *n* for each device operating at 5 different transmission power levels in environment 1 and environment 2 are shown in Table 4. It is interesting to note in the results in Table 4 that the path loss exponent increases as we progress towards the low transmission power levels. This indicates that the increase in the instability of the RSS from high to low transmission power level.

**Figure 8:**
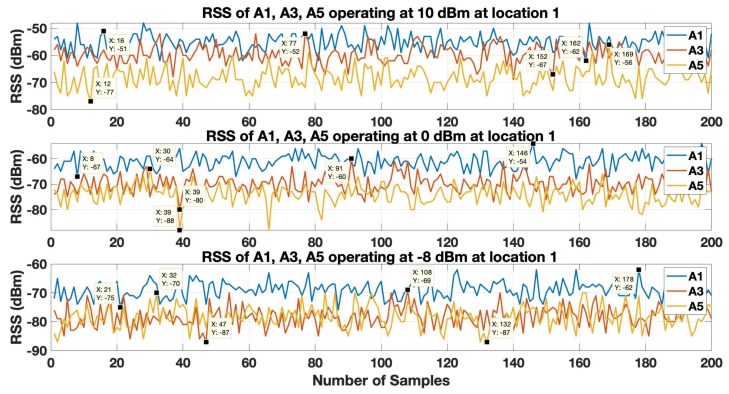
RSS of anchors $A_{1}$, $A_{3}$, and $A_{5}$ operating at 10 dBm, 0 dBm and −8 dBm at Location 1 in Environment 1.

**Figure 9:**
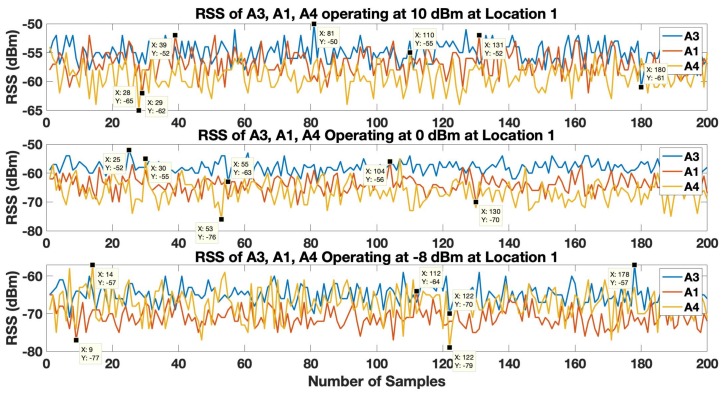
RSS of anchors $A_{3}$, $A_{1}$, and $A_{4}$ operating at 10 dBm, 0 dBm and −8 dBm at Location 1 in Environment 2.

In order to estimate the location coordinates, the tag controller initiates the localization process, which is a trilateration based localization model. The first step in the trilateration based localization model is to translate the RSS of the anchors received by the tag to the corresponding distance values with the help of the LDPL model as given by Equation (2). At each of the 3 locations, the tag is able to receive beacons of all 6 anchors. However, the 3 closest anchors are used to estimate the location of the tag. The RSS of the 3 closest anchors operating with highest (10 dBm), default (0 dBm) and lowest (−8 dBm) transmission power level, at location 1 is shown in Figure 8 and Figure 9. The strongest RSS is of anchor $A_{1}$ in Figure 8. Anchor $A_{1}$ fluctuates or varies from an approximate range of −48 dBm to −62 dBm, −54 dBm to −67 dBm and −62 dBm −75 dBm when it operates at transmission power levels of 10 dBm, 0 dBm and −8 dBm respectively. Similarly, the RSS of anchor $A_{3}$ and anchor $A_{5}$ varies at an approximate range −52 dBm to −67 dBm and −56 dBm to −77 dBm at transmission power level of 10 dBm, in range of −60 dBm to −80 dBm and −64 dBm to −88 dBm at transmission power level of 0 dBm and in the range of −69 dBm to −87 dBm and −70 dBm to −87 dBm at transmission power level of −8 dBm. From these observations it can be clearly observed that the RSS of anchor $A_{1}$ is dominant compare the RSS of the other two anchors i.e.$A_{3}$ and $A_{5}$. Also, the RSS of anchors $A_{3}$ and $A_{5}$ fluctuate a lot more in comparison to the RSS variation of anchor $A_{1}$.

In Figure 9, the RSS of the anchor $A_{3}$ is dominant compared to the RSS of anchor $A_{1}$ and anchor $A_{4}$. The RSS of the anchor $A_{3}$ varies in an approximate range of −50 dBm to −61 dBm, −52 dBm to −63 dBm and −57 dBm to −70 dBm when it operates at the transmission power levels of 10 dBm, 0 dBm and -8 dBm respectively. Similarly, the RSS of anchor $A_{1}$ and anchor $A_{4}$ varies at an approximate range −52 dBm to −62 dBm and −55 dBm to −65 dBm at the transmission power level of 10 dBm, in range of −56 dBm to −70 dBm and −55 dBm to −76 dBm at transmission power level of 0 dBm and in the range of −64 dBm to −77 dBm and −57 dBm and −79 dBm at transmission power level of −8 dBm. These results clearly show that the RSS of anchor $A_{3}$ is more strong in comparison to the RSS of the other two anchors i.e.$A_{1}$ and $A_{4}$. Also, the RSS of anchors $A_{3}$ and $A_{5}$ fluctuate a lot more in comparison to the RSS variation of anchor $A_{3}$.

The tag controller initiates the localization process. Both the CA and MMSE, trilateration methods are used to calculate the localization accuracy and localization precision. Their effects on the localization accuracy and localization precision are discussed in the next section.

#### 4.4.2. Estimating the Localization Accuracy and Localization Precision with Non-Filter Base Approach

The location of each tag, along with localization accuracy and precision were computed by using the trilateration based localization model which a non-filter based approach as shown in Figure 3. The methodology followed to calculate the results are as follows:

- Initially, 200 samples (corresponds to 1 min of data) of each anchor are collected. Based on the strongest RSS 3 anchors are selected with reference to the tag locations in both environments.
- The average RSS is calculated from the RSS sample vector. This average RSS is used to estimate the location coordinates of the tag by using trilateration based localization model with CA and MMSE method by using Equations (4) and (5).
- Localization Error: To estimate the localization error, the resultant estimate with CA method and MMSE method is used to calculate the localization error by using Equation (6). These resultant errors computed for each 3 tag location for all 5 transmission power level configurations in environment 1 and environment 2 are listed in Table 5 and Table 6.
- Each sample of the RSS sample vector is used to estimate the location coordinates of the tags with trilateration based localization model with CA and MMSE method by using Equations (4) and (5).
- Localization Precision: To estimate the localization precision, each new estimate achieved with CA method and MMSE method is then used to calculate the localization precision by using Equation (7). The localization precision results for each tag location for all 5 transmission power level configurations in environment 1 and environment 2 are listed in Table 5 and Table 6.

The results of the localization accuracy and localization precision for the trilateration based localization with CA method and MMSE method calculated in the two environments is shown in Table 5 and Table 6. The results are obtained in orderly fashion i.e., all BLE devices are configured to transmit with same transmission power level and approximately 200 RSS samples of all 6 anchors, are collected at each of the target locations. Then new configuration is made by changing the transmission power for all the BLE devices. In this way, the experiments are repeated 5 times with 5 different transmission power settings and the best results are reported in Table 5 and Table 6. The results are obtained for high (10 dBm and 4 dBm), default (0 dBm) and low transmission power levels (−4 dBm and −8 dBm). It can be observed from the results shown in Table 5 and Table 6 that localization accuracy is high at the highest transmission power level for all the locations in both indoor environments. The localization accuracy tends to decrease as the anchors operate from high to low transmission power levels for all the locations. In environment 1, on average, MMSE method results in an improvement of 0.5 m in localization accuracy compared to the results of the CA method. A similar trend can be observed from the results of localization precision. At high transmission power levels, the localization precision is marginally higher which tends to get worse as the anchors operate at low transmission power levels. Trilateration based localization with MMSE method results in an average of 3% improvement in localization precision compared to localization precision achieved by trilateration based localization with CA method. Following the same pattern, in environment 2, the MMSE method results in average improvement of 0.3 m in localization accuracy and an approximate improvement of 4% in localization precision in comparison to the results of CA method.

The reason for such results is the change in the RSS over time. If the RSS changes, the localization accuracy and localization precision will also change. A highly unstable RSS can result in large localization errors and low localization precision. In highly cluttered and dense environments, such as the ones considered in Figure 4 and Figure 5, it is highly likely that the RSS will vary and fluctuate. As localization accuracy depends on the correctness of the estimated location, a highly unstable and fluctuating RSS can lead to a faulty localization. That means that if the RSS sample vector is unstable, the LDPL model will result in incorrect distance values, which will negatively influence the location estimation with large localization errors. Similarly, an unstable RSS sample vector will contain a lot of deviated samples. If such RSS sample is used for location estimation, the resultant localization precision will be low.

Out of all these factors that affect localization accuracy and localization precision, the premier factor is the instability of the RSS due to outliers created by the multi-path interference. This is because of the dynamic nature and clutter present in the indoor environment. Movement in an indoor environment splits the wireless signal transmitted from an anchor and the clutter simple add to the cause, which makes the RSS to decay even more. Furthermore, with the decrease in the transmission power level of the anchors, the RSS also tends to decrease which makes RSS even more unstable. The instability of RSS leads to an incorrect translation of the distance from the LDPL model, thus resulting in an incorrect distance values. The incorrect distance values are taken by the localization algorithms, which results in incorrect estimations of the location coordinates of the tag, thus resulting in low localization accuracy and poor localization precision.

From the results shown in Table 5 and Table 6 it is quite clear that when anchors operate at a high transmission power level, the BLE based WILS results in reasonable localization accuracy with a marginal localization precision with a trilateration algorithm based on CA. But as we proceed towards low transmission power levels, the localization accuracy and localization precision decrease. The same pattern is followed by the trilateration algorithm based on MMSE. However, due to its error minimizing approach, the localization accuracy and localization precision is better compared to the trilateration algorithm based on CA.

To address this issue, it is important that these outliers and random variations should be removed first and RSS should be filtered before the LDPL model translates the RSS samples to distance values. By filtering, the RSS sample vector should be less fluctuating and shall not deviate a lot from their actual values. In this regard, we extend the simple trilateration based localization model in which we introduce and investigate the effect of using two filters, i.e., LPF and KF to deal with the noise effect in an RSS. The extended trilateration based localization model is discussed in the next section.

## 5. Extended Trilateration Based Localization Model

**Figure 10:**
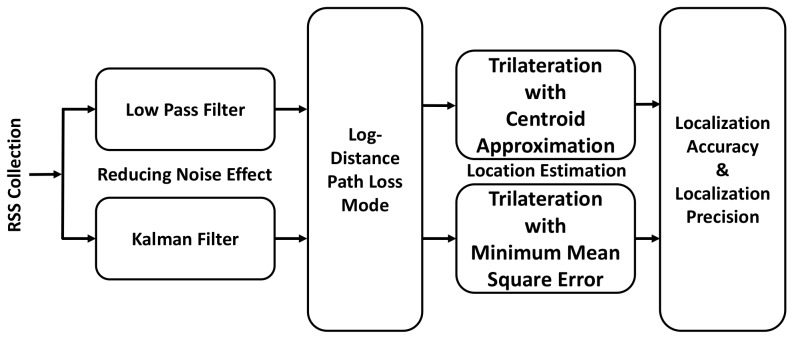
Proposed BLE based WILS Model.

In an extended trilateration based localization model, two filtering techniques i.e., LPF and KF are introduced. These filters are used to stabilize the RSS by removing the noise factors and refine the RSS sample vector. The refined RSS sample vector is used to derive the corresponding distance values by the LDPL model in which the path loss exponent *n* is recomputed. The latter results are used for the trilateration localization algorithm to estimate the location of the tags. The extended trilateration based localization model is shown in Figure 10. In the following section, LPF and KF are explained in detail.

### 5.1. Low Pass Filter

LPF is used to remove the outliers thus reducing the RSS variations [67]. Unlike traditional moving average filters, a low pass filter takes in consideration of the previous sample of the RSS along with the new incoming RSS measurement and reduces the effect of noise by scaling it with a given weight α [68]. Mathematically, an LPF is given by Equation (8) [68].

(8) $$R\hat{S}S_{\tau }=\alpha {R\hat{S}S}_{\tau -1}+\left(1-\alpha \right)RSS_{\tau }$$

where $RSS_{\tau }$ is the RSS sample vector recorded at the time τ(s), α is a weight that ranges between 0–1 and $R\hat{S}S_{\tau }$ is the new RSS sample after the filter is applied. We implemented LPF with α=0.8. The LPF is applied on the RSS of the anchors operating at 5 transmission power levels as listed in Table 1. After filtration, the new refined RSS sample vector is provided as an input to the LDPL model and trilateration localization algorithm for RSS-Distance mapping and location estimation respectively.

### 5.2. Kalman Filter

One of the finest filtering techniques commonly used to remove noise effects from RSS is the KF. In our case, we propose to use a 1-dimensional KF as our objective is to remove the unwanted noisy samples from a stream of incoming RSS samples. In order to implement the KF, there are certain filter parameters that are predetermined from the assumption model. Since a 1-dimensional KF is used over an RSS sample vector that may consist of some random variations, the transition matrix *F* and the measurement matrix *H* is assumed and set to be 1. Since there is no external control input, the control matrix $B\times u_{\tau }$ is set to zero. With these assumptions, the prediction and update phase of the 1-dimensional KF can be represented by the following set of equations [49]:

- Prediction Stage
  (9) $$R\hat{S}S_{\tau }^{-}=RSS_{\tau -1},$$
  (10) $$P_{\tau }^{-}=P_{\tau -1}+Q$$
- Update Stage
  (11) $$K_{\tau }=\frac{P_{\tau -1}^{-}}{P_{\tau -1}^{-}+R}$$
  (12) $$R\hat{S}S_{\tau }^{-}=R\hat{S}S_{\tau }^{-}+K_{\tau }\left(z_{\tau }-R\hat{S}S_{\tau }^{-}\right)$$
  (13) $$P_{\tau }=P_{\tau }^{-}\left(1-K_{\tau }\right)$$

The initial values are set to $R\hat{S}S_{\tau }^{-}$ = 0 and $P_{\tau }^{-}$ = 0. The values for *Q* = 0.055 and *R* = 1.1 are determined experimentally. The KF is applied to all the anchors operating at different transmission power levels, i.e., 10 dBm to −8 dBm. The resultant filtered RSS sample vector is then provided as input to the LDPL model and to the localization algorithms for RSS distance mapping and for location estimation as shown in Figure 10.

## 6. Experiments and Results

In this section, we present the set of experiments that we conducted to assess the BLE based WILS and compare the results based on the different techniques that were discussed in the above sections. We conducted experiments in the two indoor environments shown in Figure 4 and Figure 5 and compare our results based on the experimental settings deployed in the two indoor environments. In the both indoor environments, a total of 6 BLE anchors were chosen to locate a tag at three different locations. In this experiment, we have used Estimote BLE beacons [69] with ibeacon configurations as anchors and a Samsung Galaxy Note 3 as a tag to record the BLE beacons. The locations of the anchors and tag, in 2-dimensional coordinates, are provided in Table 3. All anchors operate with same transmission power level. However, we configure the transmission power level sequentially from 10 dBm to −8 dBm. For each configuration (let say when all anchors operate at 10 dBm) we conduct an experiment and collect 200 samples of RSS data of all anchors at all 3 target locations in two different environments as shown in Figure 4 and Figure 5. In a similar manner, we will conduct experiments for the remaining 4 configurations, repeat the experiments and compare the results. Initially, we will assess the effect of LPF and KF techniques over RSS at multiple transmission power levels. Then we will estimate the location of tag by using filter-based trilateration with CA & MMSE methods and present comparative analysis based on the estimated localization accuracy and localization precision respectively.

### 6.1. Effects on RSS

**Figure 11:**
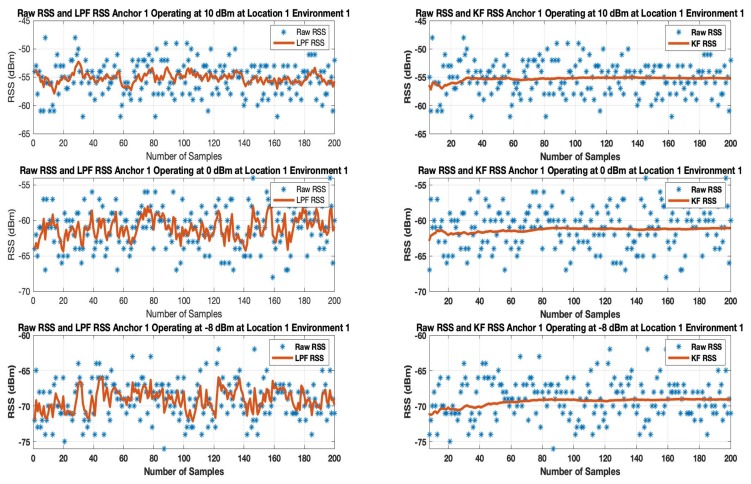
Result of LPF and KF on Anchor $A_{1}$ RSS operating at 10 dBm, 0 dBm and −8 dBm at Location 1 in Environment 1.

**Figure 12:**
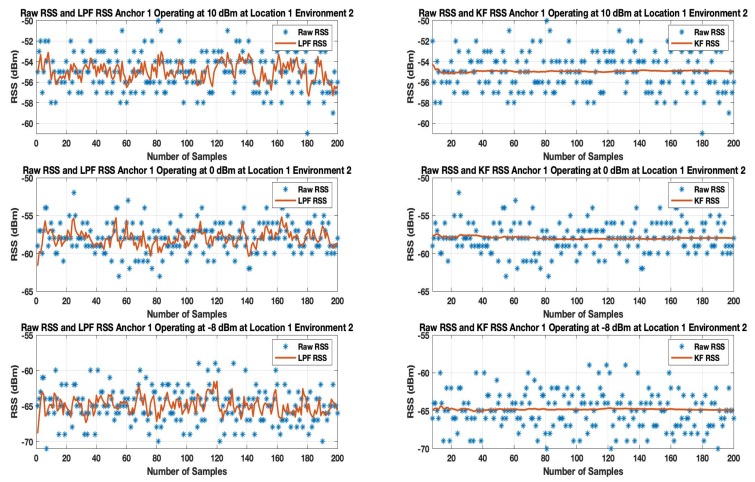
Result of LPF and KF on Anchor $A_{1}$ RSS operating at 10 dBm, 0 dBm and −8 dBm at Location 1 in Environment 2.

Initially, at each tag location 200 samples (approximately 1 min of datum) were recorded by the tag of all the anchors operating at multiple transmission power levels. LPF and KF were applied to the RSS datum. Figure 11 and Figure 12 show the results when LPF and KF are applied on anchor 1 operating on the highest (10 dBm), default (0 dBm) and the lowest (−8 dBm) transmission power level in environment 1 and environment 2.

The results show the random variation of the unfiltered BLE RSS of anchor $A_{1}$ increases as the BLE device operates from 10 dBm to -8 dBm for both indoor environments. When an LPF is applied to a BLE operating at 10 dBm as shown in Figure 11 and Figure 12, the result shows a reasonable smooth RSS. However, LPF struggles to maintain the smooth RSS, as a BLE operates from a high to low transmission power level. This is because LPF fails to map the deviated samples of RSS to their actual value of the RSS samples. A similar trend is observed in an environment as shown in Figure 12 in which LPF fails to be absolute resilient against a much deviated RSS, especially when a BLE based WILS operates at low transmission power levels. In comparison to LPF, the performance of KF in removing the effect of deviated RSS samples and smoothing the RSS sample vector outperforms the LPF as shown in Figure 11 and Figure 12. Whereas, based on the initial setting, KF converges quickly (in 1 s to 2 s). This results in extremely smooth RSS in both environments for devices operating at multiple transmission power levels.

In conclusion, both filters are able to remove the random variations from the RSS. However, KF is proved to be more effective in comparison to LPF. Thus, in terms of smoothing, KF appears to be smoother than LPF. For localization, filtered RSS streams are most likely to result in much stable localization accuracy and precision. Therefore, both filters, i.e., LPF and KF, are applied to the 3 anchors with the strongest RSS.

### 6.2. Estimating the Localization Accuracy and Localization Precision with Filter Based Approach

The filtered stream of RSS samples can now be used to estimate the location of the tag. The filters allow the RSS to be smooth by removing the random fluctuations as much as possible. This stream of filtered RSS is used to estimate the location of the tag with a filter based trilateration localization algorithm with CA and MMSE methods. The methodology used to achieve the results is defined in Section 4.4.2 in which the unfiltered RSS sample vector (i.e., 200 RSS samples corresponding to 1 min of data) is first filtered through LPF filter and KF filter. This filtered stream of RSS sample vector is used to achieve localization accuracy and localization precision. The experiments are repeated 5 times with 5 different transmission power settings at all tag locations in the two environments. The best results with filter-based approach are reported in Table 7 and Table 8. The localization accuracy and localization precision achieved for each transmission power level are shown in their respective columns.

The results show an improvement in the overall localization accuracy and localization precision at all transmission power levels with filter-based approach. A significant improvement can be observed in the localization accuracy and localization precision for the anchors that operate at low transmission power levels, i.e., −4 dBm and −8 dBm at all 3 tag locations. At a low transmission power level, the RSS tends to vary more compared to RSS at high transmission power levels. This change in the RSS sample stream causes large deviations that result in large errors.

**Figure 13:**
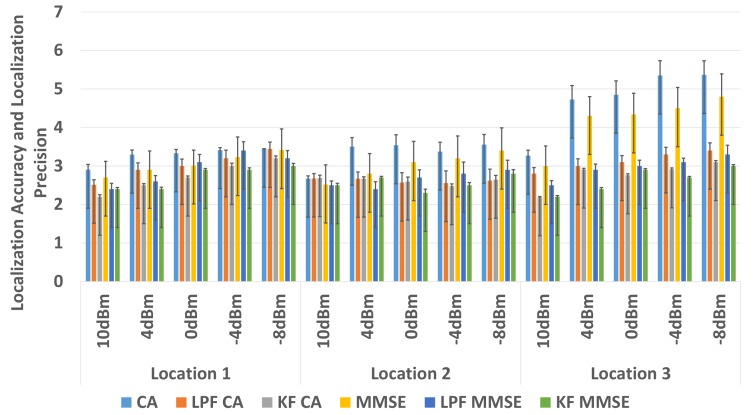
Comparison in Environment 1.

**Figure 14:**
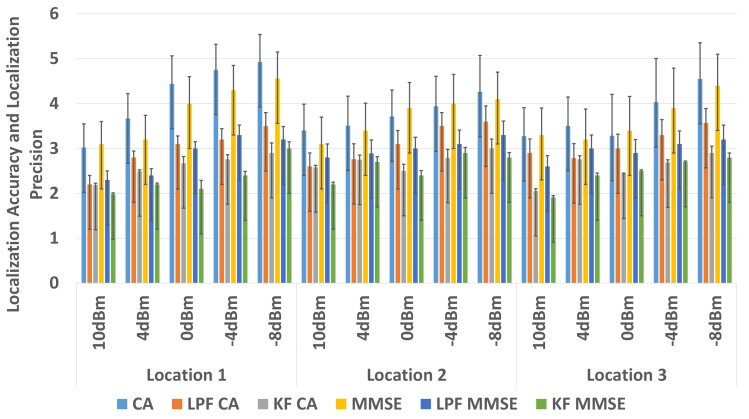
Comparison in Environment 2.

In comparison to the non-filter based approach, an average of 0.8 m of improvement is observed in localization accuracy and an approximately 36% improvement in localization precision of the WILS is observed when LPF using CA method for BLE based WILS in environment 1. An average of 1.2 m of improvement is observed in localization accuracy and an approximately 38% improvement in localization precision by the using CA method in environment 2. An average of 1.3 m improvement in localization accuracy and approximately 50% improvement in localization precision is observed with KF using CA method in environment 1. And an average of 1.4 m improvement in localization accuracy and approximately 56% improvement in localization precision is observed with KF with CA method in environment 2. With the MMSE method, an average of 0.3 m of improvement is observed in localization accuracy and an approximately 33% improvement in localization precision of the WILS is observed by using LPF in BLE based WILS in environment 1. An average of 1.4 m of improvement is observed in localization accuracy and an approximately 33.5% improvement in localization precision of the WILS is observed by using LPF in BLE based WILS for environment 2. Similarly, An average of 1 m improvement in localization accuracy and approximately 46% improvement in localization precision is observed with KF using MMSE method in BLE based WILS for environment 1. And an average of 1.4 m improvement in localization accuracy and approximately 54% improvement in localization precision is observed by using KF with MMSE method in BLE based WILS for environment 2. From the comparative analysis as shown in Figure 13 and Figure 14, it is concluded KF based MMSE method, outperforms all other methods with different transmission power levels, at all 3 locations in the two indoor environments.

Therefore, in comparison to LPF based trilateration localization with CA, the performance of a KF based trilateration localization with MMSE is far better. An average of 0.5 m improvement in localization accuracy and 10% improvement in localization precision is observed with a KF based trilateration localization with MMSE in both environments with BLE based WILS operating at multiple transmission power levels.

### 6.3. Comparison with the State-of-the-Art

In this section, we shall compare our results with results of the two relevant works i.e., [24,28], present in the literature. The best result reported in [24] is a localization accuracy of 2 m with matching precision (localization precision) of approximately 86.74% by using 5 BLE anchors with Mode-KNN based signal pattern matching method. Whereas, the best results reported in [28] is a localization accuracy of 2.8 m only, achieved by using 3 BLE anchors with minimum signal replacement based signal patter matching method.

In comparison to the results reported above, we have been able to achieve localization accuracy is approximately 2.2 m meters with a localization precision 95% by using 3 BLE anchors with KF based trilateration method.

## 7. Conclusions and Future Work

BLE based WILS with high accuracy and high precision is extremely important to improve the location-based BLE IoT applications. In this paper, we investigated the problem of RSS variation incurred by the use of multiple transmission power feature in BLE based WILS. To highlight this problem, we deployed BLE based WILS in two different kinds of indoor environments as shown in Figure 4 and Figure 5 that consisted of 6 anchors to estimate at tag coordinates at 3 different tag locations with a trilateration based localization model. It was observed that the RSS, localization accuracy and localization precision tends to decrease as the anchors operate from high to low transmission power levels. We initially used a non-filter based approach in which we compared the results of two commonly used trilateration methods i.e., CA method and MMSE method. Furthermore, we analyzed the results and concluded that the trilateration based localization model with MMSE outperformed the CA method in terms of localization accuracy and localization precision in both indoor environments.

To improve the overall localization accuracy and localization precision of BLE based WILS, we further investigated the effects of the use of two filters i.e., LPF and KF in trilateration based localization model with CA and MMSE methods. In comparison to the non-filter based approach, we observed a great improvement in localization accuracy and localization precision with filter-based approach in the two indoor environments. We compared the results of LPF and KF as shown Figure 13 and Figure 14. We observed the results obtained with the use of KF in trilateration based localization with MMSE were far better than the results obtained with LPF in the trilateration based localization model with CA and MMSE methods. In conclusion, the use of KF in trilateration based localization model with MMSE method proved more effective in eradicating random variations in RSS with the change in multiple transmission power levels, thus resulting in a BLE based WILS with high accuracy and high precision.

In future, we are focusing to address the KF error drift issue. In our case, whenever KF is invoked, it starts from the initial values (reset each time to 0) which is one method to address this problem. Another approach is that the KF starts with an initial value of the RSS for each anchor to help in quick convergence (less than 1–2 s). However, there are two problems in this approach, (1) the setting initial RSS values for each anchor add extra workload and (2) with each filter error is added with the initial settings (error drift issue) which can drift the KF result overtime. Also, the problem of BLE RSS signal variation caused by the change in transmission power levels needs to be investigated with semi-dynamic and dynamic targets in an indoor environment. In this regard, it would be interesting to see the impact of the Extended Kalman Filter (EKF) and Particle Filter (PF). Furthermore, these filters can also be used in conjunction with fingerprinting-based localization algorithms. We could also consider security of ranging as this has become an interesting requirement in some applications [70]. Thus, we plan to extend our work in these dimensions as our future work.

## Figures and Tables

**Table 1:** List of transmission power levels and their transmission ranges [56] (©IEEE 2018).

Power Levels (*l*)	Transmission Power (ρ) (dBm)	Range (m)
0	10 dBm	200
1	4 dBm	70
2	0 dBm	50
3	−4 dBm	40
4	−8 dBm	30
5	−12 dBm	15
6	−16 dBm	7.0
7	−20 dBm	3.5
8	−40 dBm	1.0

**Table 2:** Symbols and Definitions.

Symbols	Definition
$A_{k}$	Set of *k* anchors
$T_{t}$	Set of *t* targets
$x_{A_{k}},y_{A_{k}}$	True coordinates of anchor
$x_{T_{t}},y_{T_{t}}$	True coordinates of tag
X=(${\hat{x}}_{T_{t}},{\hat{y}}_{T_{t}}$)	Estimated coordinates of tag
$d_{A_{k},T_{t}}$	Distance between an anchor $A_{k}$ and a tag $T_{t}$
RSS	Raw RSS sample vector
$RSS(d_{0})$	RSS collected at distance $d_{0}$ = 1 m
$R\hat{S}S$	Filtered RSS sample vector
*n*	Path loss exponent
UUID	Universal Unique Identifier
$\tau_{adv}$	Advertising interval
$\tau_{scan}$	Scanning interval
${\rho_{l}}^{\tau }$	Transmission power level l at time τ
τ	Time instance (s)
*S*	Anchor coordinate matrix
*b*	Anchor distance-coordinate matrix
$\varepsilon (T_{t})$	Localization accuracy or Localization error (m) of tag $T_{t}$
$\sigma (T_{t})$	Localization Precision of tag $T_{t}$, ranges from [0–1]
α	LPF weight, ranges from [0–1]
*F*	KF transition matrix
*H*	KF measurement matrix
*B*	KF control matrix
$u_{\tau -1}$	KF control input
$P_{\tau }^{-}$	KF New prediction
$P_{\tau -1}$	KF previous prediction
$P_{\tau }$	KF update in the prediction
$K_{\tau }$	KF kalman gain
*Q*	KF process noise matrix
*R*	KF measurement noise matrix

**Table 3:** Locations of Anchors and Tags.

Environment 1				Environment 2			
**Anchors**	**Locations (m)**	**Tag**	**Locations (m)**	**Anchors**	**Locations (m)**	**Tag**	**Locations (m)**
$A_{1}$	(1.5, 1.5)	$T_{1}$	(4, 1.5)	$A_{1}$	(1, 0)	$T_{1}$	(3, 4)
$A_{2}$	(12, 1.5)	$T_{2}$	(10, 4.5)	$A_{2}$	(7, 0)	$T_{2}$	(6, 6)
$A_{3}$	(0, 3.5)	$T_{3}$	(3, 7.5)	$A_{3}$	(0, 6)	$T_{3}$	(1.5, 9)
$A_{4}$	(13.5, 3.5)			$A_{4}$	(8, 7)		
$A_{5}$	(1.5, 9)			$A_{5}$	(2, 12)		
$A_{6}$	(12, 9)			$A_{6}$	(5, 12)		

**Table 4:** Path loss exponent in two different indoor environments.

	High Transmission Power			Default Transmission Power		Low Transmission Power	
	10 dBm	4 dBm		0 dBm		−4 dBm	−8 dBm
Environment 1	n = 2.2	n = 3		n = 2.8		n = 2.9	n = 2.9
Environment 2	n = 2.2	n = 2.8		n = 3		n = 3	n = 3

**Table 5:** Localization Accuracy and Localization Precision in Environment 1.

Environment 1	10 dBm		4 dBm		0 dBm		−4 dBm		−8 dBm	
Location 1	CA	MMSE	CA	MMSE	CA	MMSE	CA	MMSE	CA	MMSE
$\varepsilon (T_{1})$ (m)	2.904	2.7	3.294	2.9	3.329	3.012	3.412	3.231	3.446	3.412
$\sigma (T_{1})$	0.49	0.42	0.51	0.49	0.43	0.4	0.59	0.521	0.601	0.55
Location 2										
$\varepsilon (T_{2})$ (m)	2.673	2.52	3.5	2.8	3.537	3.1	3.375	3.2	3.554	3.4
$\sigma (T_{2})$	0.55	0.51	0.545	0.52	0.56	0.54	0.646	0.58	0.620	0.59
Location 3										
$\varepsilon (T_{3})$ (m)	3.267	3	4.725	4.3	4.849	4.34	5.350	4.5	5.364	4.8
$\sigma (T_{3})$	0.49	0.52	0.51	0.5	0.55	0.55	0.56	0.54	0.61	0.59

**Table 6:** Localization Accuracy and Localization Precision in Environment 2.

Environment 2	10 dBm		4 dBm		0 dBm		−4 dBm		−8 dBm	
Location 1	CA	MMSE	CA	MMSE	CA	MMSE	CA	MMSE	CA	MMSE
$\varepsilon (T_{1})$ (m)	3.024	3.1	3.670	3.2	4.440	4	4.754	4.3	4.927	4.56
$\sigma (T_{1})$	0.524	0.5	0.551	0.54	0.624	0.6	0.569	0.55	0.620	0.59
Location 2										
$\varepsilon (T_{2})$ (m)	3.403	3.1	3.508	3.4	3.712	3.9	3.940	4	4.263	4.1
$\sigma (T_{2})$	0.583	0.6	0.656	0.61	0.590	0.57	0.670	0.65	0.811	0.6
Location 3										
$\varepsilon (T_{3})$ (m)	3.276	3.3	3.505	3.2	3.285	3.4	4.034	3.9	4.550	4.4
$\sigma (T_{3})$	0.633	0.6	0.643	0.68	0.920	0.76	0.970	0.89	0.804	0.7

**Table 7:** Localization Accuracy and Localization Precision by using LPF at 3 Tag Locations in Environment 1 and Environment 2.

	Environment 1									
	**10 dBm**		**4 dBm**		**0 dBm**		−4 **dBm**		−8 **dBm**	
Location 1	CA	MMSE	CA	MMSE	CA	MMSE	CA	MMSE	CA	MMSE
$\varepsilon (T_{1})$ (m)	2.513	2.4	2.9	2.6	3	3.1	3.2	3.4	3.449	3.2
$\sigma (T_{1})$	0.129	0.15	0.181	0.15	0.178	0.2	0.210	0.23	0.177	0.2
Location 2										
$\varepsilon (T_{2})$ (m)	2.674	2.5	2.67	2.4	2.570	2.7	2.556	2.8	2.619	2.9
$\sigma (T_{2})$	0.128	0.11	0.176	0.19	0.257	0.2	0.317	0.3	0.298	0.25
Location 3										
$\varepsilon (T_{3})$ (m)	2.8	2.5	3	2.9	3.1	3	3.3	3.1	3.4	3.3
$\sigma (T_{3})$	0.158	0.12	0.185	0.15	0.170	0.15	0.185	0.14	0.2	0.24
	**Environment 2**									
	**10 dBm**		**4 dBm**		**0 dBm**		−4 **dBm**		−8 **dBm**	
Location 1	CA	MMSE	CA	MMSE	CA	MMSE	CA	MMSE	CA	MMSE
$\varepsilon (T_{1})$ (m)	2.2	2.3	2.8	2.4	3.1	3	3.2	3.3	3.5	3.2
$\sigma (T_{1})$	0.199	0.2	0.144	0.15	0.18	0.15	0.24	0.22	0.3	0.29
Location 2										
$\varepsilon (T_{2})$ (m)	2.602	2.8	2.763	2.89	3.1	3	3.5	3.1	3.6	3.3
$\sigma (T_{2})$	0.210	0.3	0.342	0.3	0.3	0.25	0.3	0.31	0.35	0.31
Location 3										
$\varepsilon (T_{3})$ (m)	2.9	2.6	2.784	3	3	2.9	3.3	3.1	3.57	3.2
$\sigma (T_{3})$	0.311	0.24	0.325	0.3	0.32	0.3	0.35	0.29	0.32	0.3

**Table 8:** Localization Accuracy and Localization Precision by using KF at 3 Tag Locations in Environment 1 and Environment 2.

	Environment 1									
	**10 dBm**		**4 dBm**		**0 dBm**		−4 **dBm**		−8 **dBm**	
Location 1	CA	MMSE	CA	MMSE	CA	MMSE	CA	MMSE	CA	MMSE
$\varepsilon (T_{1})$ (m)	2.2	1.7	2.3	2.2	2.7	2.4	3	2.5	3.2	2.65
$\sigma (T_{1})$	0.052	0.041	0.042	0.05	0.0401	0.034	0.071	0.052	0.060	0.06
Location 2										
$\varepsilon (T_{2})$ (m)	2.186	1.9	2.471	2.1	2.596	2.3	2.677	2.5	2.643	2.8
$\sigma (T_{2})$	0.075	0.05	0.046	0.031	0.116	0.098	0.0585	0.07	0.112	0.104
Location 3										
$\varepsilon (T_{3})$ (m)	2.089	1.8	2.308	2.023	2.758	2.2	2.914	2.4	3.1	2.8
$\sigma (T_{3})$	0.019	0.03	0.030	0.040	0.039	0.031	0.036	0.029	0.038	0.035
	**Environment 2**									
	**10 dBm**		**4 dBm**		**0 dBm**		−4 **dBm**		−8 **dBm**	
Location 1	CA	MMSE	CA	MMSE	CA	MMSE	CA	MMSE	CA	MMSE
$\varepsilon (T_{1})$ (m)	2.189	1.98	2.492	2.1	2.67	2.2	2.76	2.4	2.9	2.5
$\sigma (T_{1})$	0.038	0.032	0.032	0.029	0.152	0.19	0.101	0.091	0.224	0.15
Location 2										
$\varepsilon (T_{2})$ (m)	2.280	2.0	2.509	2.3	2.5	2.4	2.89	2.6	3	2.54
$\sigma (T_{2})$	0.044	0.05	0.102	0.12	0.15	0.109	0.19	0.123	0.21	0.11
Location 3										
$\varepsilon (T_{3})$ (m)	2.053	1.91	2.359	2.0	2.44	2.3	2.69	2.4	2.9	2.50
$\sigma (T_{3})$	0.051	0.04	0.080	0.052	0.012	0.024	0.057	0.02	0.15	0.1

## Abbreviations

The following abbreviations are used in this manuscript:

IoT	Internet of Things
BLE	Bluetooth Low Energy
WiFi	Wireless Fidelity
RFID	Radio Frequency IDentification
UWB	Ultra Wide Band
WILS	Wireless Indoor Localization System
BER	Bit Error Rate
CA	Centeroid Approximation
MMSE	Minimum Mean Square Error Method
LPF	Low Pass Filter
KF	Kalman Filter
EKF	Extended Kalman Filter
PF	Particle Filter
RSS	Received Signal Strength
dBm	Decibel-miliwatts
ms	Milli Second
m	Meters
s	Seconds
